# Additions to Dictyosporiaceae: *Neoxylochrysis typhicola* comb. et gen. nov., Two New Species and Four New Host Records from Medicinal Plants in Southwestern China

**DOI:** 10.3390/jof10120872

**Published:** 2024-12-16

**Authors:** Na Wu, Hong-Zhi Du, Kandawatte Wedaralalage Thilini Chethana, Kitiphong Khongphinitbunjong, Sajeewa S. N. Maharachchikumbura, Kevin D. Hyde, Jian-Kui Liu

**Affiliations:** 1 School of Life Science and Technology, Center for Informational Biology, University of Electronic Science and Technology of China, Chengdu 611731, China; wuna220@gmail.com (N.W.); hongzhi_du1012cc@163.com (H.-Z.D.); sajeewa83@yahoo.com (S.S.N.M.); 2 Center of Excellence in Fungal Research, Mae Fah Luang University, Chiang Rai 57100, Thailand; tchethi@yahoo.com (K.W.T.C.); kdhyde3@gmail.com (K.D.H.); 3 School of Science, Mae Fah Luang University, Chiang Rai 57100, Thailand; kitiphong.kho@mfu.ac.th; 4 School of Pharmacy, Guizhou University of Traditional Chinese Medicine, Guiyang 550025, China; 5 Department of Entomology and Plant Pathology, Faculty of Agriculture, Chiang Mai University, Chiang Mai 50200, Thailand

**Keywords:** Dothideomycetes, multi-locus phylogeny, *Pseudocoleophoma*, taxonomy

## Abstract

Medicinal plants serve as vital resources for preventing and treating diseases, with their flowers, fruits, leaves, roots, or entire plants being utilized in the pharmaceutical industry or as direct therapeutic agents. During our investigation of microfungi associated with medicinal plants in Guizhou and Sichuan Provinces, China, several asexual and sexual fungal morphs were collected. Multi-locus phylogenetic analysis based on combined ITS, LSU, SSU and *TEF1-α* datasets revealed that these taxa are related to the family Dictyosporiaceae. Morphological characteristics, along with multi-locus phylogenetic analysis, supported the establishment of *Dictyocheirospora alangii* sp. nov. and *Pseudocoleophoma rosae* sp. nov., as well as the introduction of a novel genus *Neoxylochrysis*, which accommodates *Neoxylochrysis typhicola* comb. nov. (≡*Pseudocoleophoma typhicola*). In addition, four new host records are introduced for *Aquadictyospora lignicola* from *Periploca forrestii*, *Dendryphiella eucalyptorum* from *Leonurus japonicus*, *Ophiopogon japonicus* and *Sambucus javanica*, *D. vinosa* from *Phytolacca americana*, and *Dictyocheirospora rotunda* from *Euonymus japonicus* and *Prinsepia utilis*. Detailed descriptions, micrographs of the new taxa and a phylogenetic tree are provided.

## 1. Introduction

The family Dictyosporiaceae, originally proposed as Dictyosporaceae by Liu et al. [1], was formally introduced by Boonmee et al. [2] within the order Pleosporales and class Dothideomycetes. The type genus of Dictyosporiaceae is *Dictyosporium* Corda, with *D. elegans* designated as the type species. This holomorphic family is globally distributed and consists of 20 genera. Most asexual morphs in this family are hyphomycetous, viz., *Aquadictyospora*, *Aquaticheirospora*, *Cheirosporium*, *Dendryphiella*, *Dictyocheirospora*, *Dictyopalmispora*, *Dictyosporium*, *Digitodesmium*, *Jalapriya*, *Neodendryphiella*, *Neodigitodesmium*, *Pseudodictyosporium* and *Vikalpa*, characterized by the production of cheiroid (digitate), multi-septate, palmate or dictyosporous, and pale brown to brown conidia [2,3,4,5,6,7]. A few genera, viz., *Immotthia*, *Pseudocoleophoma*, *Pseudoconiothyrium*, *Pseudocyclothyriella*, *Sajamaea* and *Verrucoccum* have coelomycetous asexual morphs [8,9,10,11,12,13]. Among these, only five genera viz., *Dictyosporium*, *Gregarithecium*, *Immotthia*, *Pseudocoleophoma* and *Verrucoccum*, are known to produce sexual morphs, which are characterized by brown to black ascomata, bitunicate, cylindric-clavate asci with a short ocular chamber, and septate, hyaline to brown, sheathed ascospores [8,9,12]. Notably, *Gregarithecium* lacks an asexual morph [9].

The genus *Pseudocoleophoma*, typified by *P. calamagrostidis* Kaz. Tanaka and K. Hiray., was established by Tanaka et al. [9] to accommodate two species, *P. calamagrostidis* and *P. polygonicola*. Fourteen *Pseudocoleophoma* species are listed in Index Fungorum (accessed September 2024), viz., *P. bauhiniae*, *P. calamagrostidis*, *P. clematidis*, *P. flavescens*, *P*. *guizhouensis*, *P*. *heteropanacicola*, *P. paraphysoidea*, *P. polygonicola*, *P. puerensis*, *P. rhapidis*, *P. rusci*, *P. typhicola*, *P. yunnanensis* and *P. zingiberacearum* [13,14]. However, *P. clematidis* was transferred to *Pseudocyclothyriella* based on morphology and multi-locus phylogenetic analysis. by Jiang et al. [13]. The sexual morph of *Pseudocoleophoma* is characterized by cylindrical to clavate asci and hyaline, fusiform, septate ascospores with an apparent sheath [15,16], while the asexual morph is characterized by hyaline, aseptate, cylindrical or oblong, smooth-walled conidia with obtuse ends [17,18,19,20].

*Aquadictyospora* was introduced by Li et al. [5] to accommodate the species, *A. lignicola*, collected from submerged decaying wood in China. Phukhamsakda et al. [21] introduced the second species, *A. clematidis*, based on morphology and phylogenetic analyses. *Aquadictyospora* is an asexual morph genus, with no sexual morph linked to it. The genus is characterized by superficial, compact, scattered, globose or subglobose, dark brown to black conidiomata, micronematous conidiophores with monoblastic conidiogenous cells, and uniformly medium brown conidia with a broadly ovate to subglobose, hyaline cell in the lower half [5,21].

*Dendryphiella* was introduced by Ranojevic [22] with the type species *D. interseminata* Bubák. This asexual genus has 20 epithets in Index Fungorum (accessed September 2024). It is characterized by macronematous, fasciculate, septate conidiophores with polytretic, verrucose conidiogenous cells enlarged at the apex, and solitary to catenate, aseptate or septate, hyaline to pale brown, thick-walled, verrucose conidia [23,24]. It is worth noting that the mode of conidiogenous cell development of *Dendryphiella* members is tretic, while other asexual morphs in the family are blastic [2,6,23]. Boonmee et al. [2] established *Dictyocheirospora* to accommodate three species, *D. bannica*, *D. rotunda* (type species) and *D. vinaya*, and transferred four species of *Dictyosporium* to *Dictyocheirospora*. Currently, 29 epithets of *Dictyocheirospora* are listed in Index Fungorum (accessed September 2024). *Dictyocheirospora* is similar to *Dictyosporium* except for the conidial arms, which are arranged differently [2,25,26,27]. The sexual stage of *Dictyocheirospora* has never been reported.

In this study, we explored the diversity and taxonomy of the family Dictyosporiaceae, focusing on species associated with medicinal plants. The taxa of Dictyosporiaceae have been subject to frequent taxonomic revisions and phylogenetic realignments, reflecting the complexity of their evolutionary relationships. By examining Dictyosporiaceae species collected from medicinal plant, we aimed to expand the understanding of their biodiversity, refine their taxonomy through multi-locus phylogenetic analyses, and assess their ecological roles in these specialized habitats. This study also contributes to the understanding of classification and biodiversity of this diverse fungal family.

## 2. Materials and Methods

### 2.1. Specimen Collection, Examination, and Single Spore Isolation

**Figure 1:**
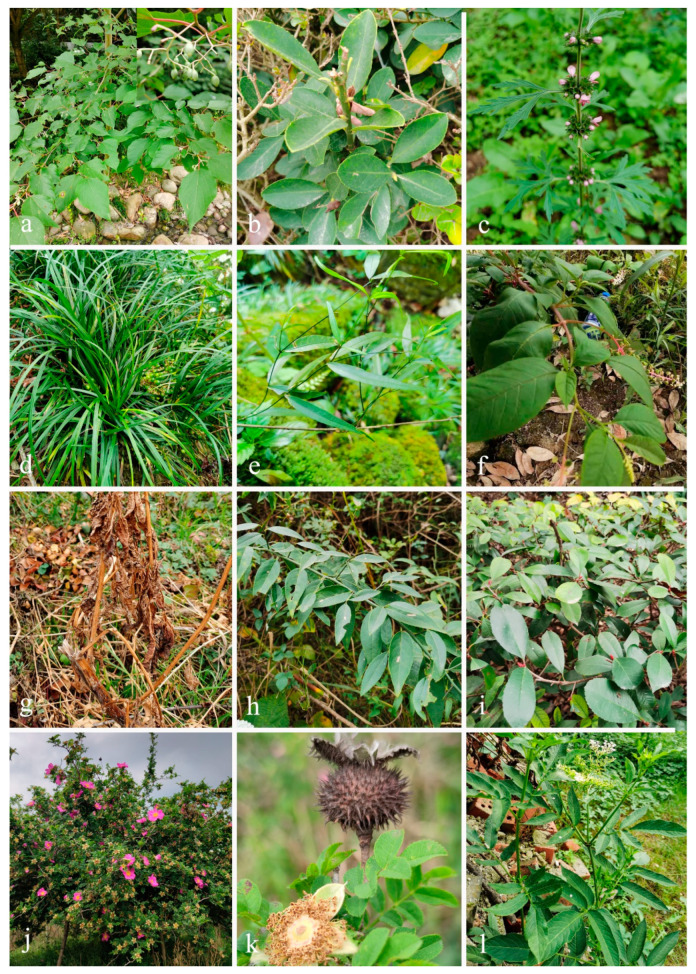
Photos of medicinal plant hosts in this study (**a**) *Alangium chinense* (Cornaceae). (**b**) *Euonymus japonicus* (Celastraceae). (**c**) *Leonurus japonicus* (Lamiaceae). (**d**) *Ophiopogon japonicus* (Asparagaceae). (**e**) *Periploca forrestii* (Apocynaceae). (**f**,**g**) *Phytolacca americana* (Phytolaccaceae). (**h**) *Prinsepia utilis* (Rosaceae). (**i**) *Rhaphiolepis indica* (Rosaceae). (**j**,**k**) *Rosa roxburghii* (Rosaceae). (**l**) *Sambucus javanica* (Viburnaceae).

The dead or decaying leaves and twigs of medicinal plants (*Alangium chinense*, *Euonymus japonicus*, *Leonurus japonicus*, *Ophiopogon japonicus*, *Periploca forrestii*, *Phytolacca americana*, *Prinsepia utilis*, *Rhaphiolepis indica*, *Rosa roxbunghii* and *Sambucus javanica*, Figure 1) were collected from Guizhou and Sichuan Provinces, China (detailed information about the collection sites is provided in the ‘Materials examined’ subsection of Section 3.2). The samples were kept in paper envelopes and brought to the laboratory following the method described in Senanayake et al. [28]. Morphological observations of fungal structures were made using a Nikon SMZ745 dissecting microscope (Nikon Corporation, Tokyo, Japan), following the method described in Chomnunti et al. [29]. Photomicrographs of the fungal specimens were captured using a Nikon Eclipse Ni-U compound microscope fitted with a Nikon DS-Ri2 digital camera (Nikon Corporation, Tokyo, Japan). Macro-morphological structures were photographed with a Nikon SMZ800N stereo microscope fitted with a Nikon DS-Fi3 microscope camera (Nikon Corporation, Tokyo, Japan). All measurements were made with the Tarosoft Image Frame Work program v. 0.97, and the photo-plates were made with Adobe Photoshop CC extended version 21.1.2. Single spore isolations were conducted in accordance with the methods described in Senanayake et al. [28]. Germinated spores were transferred to fresh potato dextrose agar (PDA), incubated at 25 °C, and the colony characteristics were observed and recorded after one week, following the method described in Rayner [30].

Herbarium specimens were deposited in the herbarium of Cryptogams, Kunming Institute of Botany, Chinese Academy Sciences (KUN-HKAS), Kunming, China and the Herbarium of University of Electronic Science and Technology (HUEST), Chengdu, China. The pure cultures were deposited in the China General Microbiological Culture Collection Centre (CGMCC), Beijing, China and the University of Electronic Science and Technology Culture Collection (UESTCC), Chengdu, China. Faces of Fungi and Index Fungorum numbers were provided for the new taxa (Index Fungorum 2024) [31].

### 2.2. DNA Extraction, PCR Amplification and Sequencing

Fresh mycelia (about 50–100 mg) were scraped using a sterilized toothpick from the margin of a colony on a PDA plate, which had been incubated at 25 °C for two to three weeks [32], and stored in 1.5 mL sterilized micro-centrifuge tubes and maintained at −20 °C for long term storage. The Trelief^TM^ Plant Genomic DNA Kit (TSINGKE Biotech, Shanghai, China) was used to extract DNA according to the manufacturer’s instructions. The obtained genomic DNA was stored in two tubes, one at 4 °C for polymerase chain reaction (PCR) amplification and the other at −20 °C for long-term storage. The internal transcribed spacer (ITS), the partial 28S large subunit rRNA (LSU), the partial 18S small subunit rRNA (SSU), and the partial translation elongation factor 1-alpha (*TEF1-α*) regions were amplified using the following primers: ITS5 and ITS4 [33] for ITS, LR0R and LR5 [34] for LSU, NS1 and NS4 [33] for SSU, and EF1-983F and EF1-2218R [35] for *TEF1-α*. The final volume (25 μL) contained 2 μL DNA, 12.5 μL PCR mix, 8.5 μL distilled water and 1 μL of each primer.

The PCR thermal cycle program of ITS, LSU, and SSU loci used the following conditions: initial denaturation at 94 °C for 3 min, followed by 40 cycles of denaturation at 94 °C for 45 s, annealing at 56 °C for 50 s, elongation at 72 °C for 1 min, and a final extension at 72 °C for 10 min. The *TEF1-α* amplification followed these conditions: initial denaturation at 94 °C for 5 min, followed by 34 cycles of denaturation at 94 °C for 30 s, annealing at 55 °C for 50 s, elongation at 72 °C for 1 min, and a final extension at 72 °C for 5 min. The products were visualized on 1% agarose gel under UV light in a Gel DocTM XR and sequenced at Sangon Biotechnology Co. (Chengdu, China).

### 2.3. Sequence Alignment and Phylogenetic Analysis

Sequences generated in this study were checked and assembled using BioEdit v. 17.0.1 [36] to ensure sequence quality. Through the BLASTn search tool on NCBI [37] (https://blast.ncbi.nlm.nih.gov/, accessed on 20 August 2023), based on newly generated ITS and LSU sequence data, we found out that our taxa were related to Dictyosporiaceae. According to the BLAST results and previous literature, appropriate sequences were downloaded from GenBank to construct phylogenetic analyses. Two isolates of *Periconia igniaria* (CBS 379.86 and CBS 845.96) were selected as the outgroup. Details of the isolates used in this study are listed in Table 1. The sequences were aligned using MAFFT v.7 online [38] (https://mafft.cbrc.jp/alignment/server/, accessed on 28 August 2023) and AliView [39], and the results were checked using BioEdit v. 17.0.1 [36] and manually edited where necessary. The concatenation of the genes was conducted using SequenceMatrix 1.8 [40]. The Nexus and Phylip files for phylogenetic analyses were obtained using AliView [39]. Phylogenetic analyses of the combined sequence data were performed using maximum likelihood (ML) and Bayesian inference (BI) methods, as detailed in Dissanayake et al. [41]. Best-fit models for BI analyses were selected using MrModeltest v. 2.2 [42]. The ML analysis was performed using RAxML GUI v. 1.3.1 [43], and the BI analysis was conducted in MrBayes v 3.2.6 [44]. Phylogenetic trees were visualized with FigTree v.1.4.4 [45] (http://tree.bio.ed.ac.uk/software/figtree/, accessed on 9 October 2023) and further edited in Adobe Illustrator 2020 (Adobe Systems, San Jose, CA, USA). The final alignment was submitted to Figshare [46] (https://figshare.com, at https://doi.org/10.6084/m9.figshare.26085910, accessed on 23 June 2024).

## 3. Results

### 3.1. Phylogenetic Analysis

**Figure 2:**
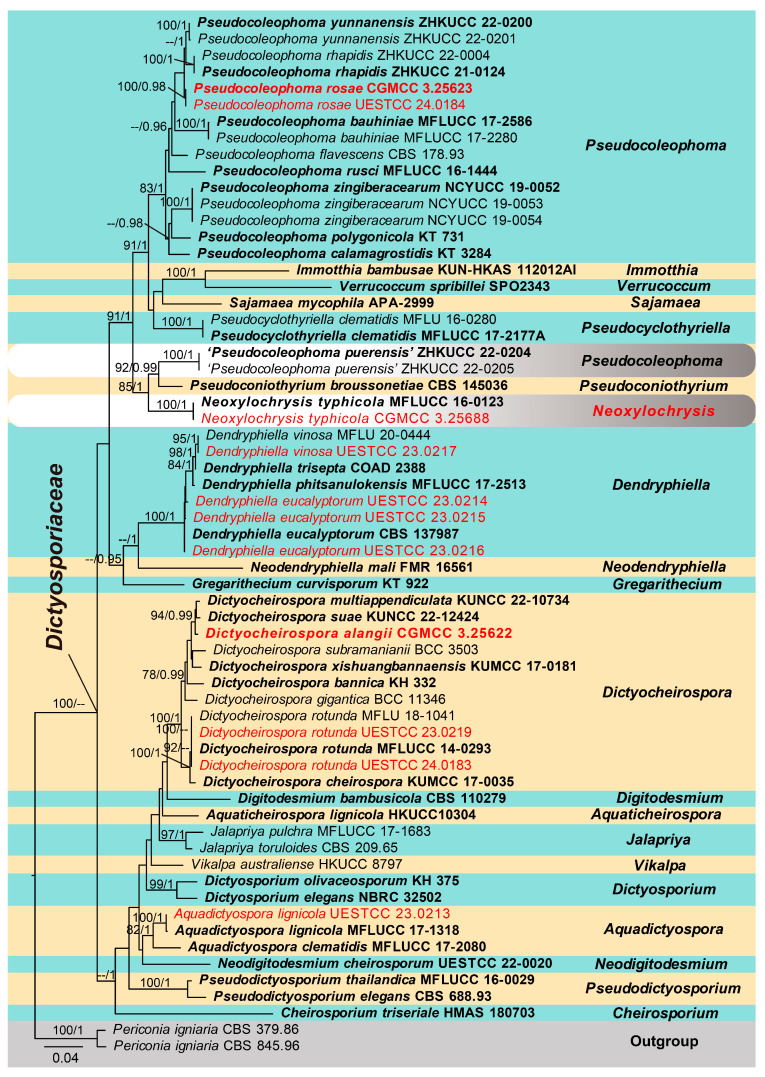
Phylogenetic tree generated from the maximum likelihood analysis based on the combined ITS, LSU, SSU and *TEF1-α* sequence data of Dictyosporiaceae. The ML (≥75%) and BI (≥95%) bootstrap supports are given near the nodes, respectively. The new isolates obtained in this study are indicated in red and ex-type strains are in bold. The tree is rooted with *Periconia igniaria* (CBS 379.86 and CBS 845.96).

The analyzed dataset was composed of the combined ITS, LSU, SSU and *TEF1-α* sequence data from 61 taxa (ingroup) with *Periconia igniaria* (CBS 379.86 and CBS 845.96) as the outgroup (Figure 2). The aligned dataset comprised 3849 characters (ITS: 574 bp, LSU: 883 bp, SSU: 1436 bp, *TEF1-α*: 956 bp) including gaps. The RAxML analysis of the combined data set yielded a best-scoring tree (Figure 2) with a final ML optimization likelihood value of −20,233.812942. RAxML and Bayesian analyses were conducted and resulted in generally congruent topologies and the familial assignment is similar to previous work [6,27].

The multi-locus phylogenetic analyses showed that eleven isolates obtained in this study were nested within the family Dictyosporiaceae, of which one isolate (UESTCC 23.0213) was identified as *Aquadictyospora lignicola*. Four isolates (UESTCC 23.0214, UESTCC 23.0215, UESTCC 23.0216 and UESTCC 23.0217) clustered with *Dendryphiella eucalyptorum* and *D. vinosa*, respectively. Three isolates belong to the genus *Dictyocheirospora*, of which UESTCC 23.0219 and UESTCC 24.0183 clustered with *Dictyocheirospora rotunda*, while the new taxon *Dictyocheirospora alangii* (CGMCC 3.25622) clustered sister to *Di. multiappendiculata* (KUNCC 22-10734) and *Di. suae* (KUNCC 22-12424) with 94% ML bootstrap support (MLBS) and 0.99 Bayesian posterior probability support (BYPP) (Figure 2). Two isolates (CGMCC 3.25623 and UESTCC 24.0184) nested within the genus *Pseudocoleophoma*, but did not cluster with any previously known species; thus, a novel species, *P. rosae*, was preliminarily identified. In addition, our isolate CGMCC 3.25688 clustered with *Neoxylochrysis typhicola* (≡*Pseudocoleophoma typhicola*) (MFLUCC 16-0123), separated from all taxa of *Pseudocoleophoma* and formed a clade basal to *P. puerensis* (ZHKUCC 22-0204, ZHKUCC 22-0205) and *Pseudoconiothyrium broussonetiae* (CBS 145036). Thus, based on current phylogenetic status, *P*. *typhicola* is transferred to the novel genus *Neoxylochrysis* as *N. typhicola*.

### 3.2. Taxonomy

**Figure 3:**
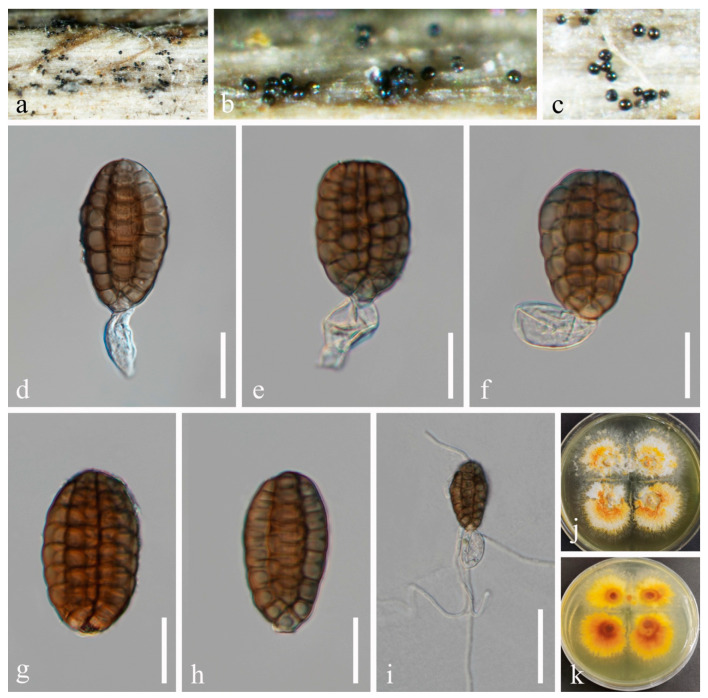
*Aquadictyospora lignicola* (HUEST 23.0213, new host record). (**a**–**c**) Colonies on a woody substrate. (**d**–**f**) Conidia with partial conidiophores. (**g**,**h**) Conidia. (**i**) Germinated conidium. (**j**,**k**) Colonies on PDA, from above (**j**), from below (**k**). Scale bars: (**d**–**h**) = 20 μm, (**i**) = 50 μm.

***Aquadictyospora lignicola*** Z.L. Luo, W.L. Li, K.D. Hyde & H.Y. Su, Mycosphere 8(10): 1591 [5] (Figure 3).

Index Fungorum number: IF553862; Faces of Fungi number: FoF03768

*Saprobic* on dead twigs of *Periploca forrestii* Schltr. Sexual morph: Not observed. Asexual morph: Hyphomycetous. *Colonies* superficial, gregarious, scattered, punctiform, sporodochial, scattered, black, velvety, glistening, orbicular. *Mycelium* mostly immersed or partly superficial, smooth, with hyaline to pale brown hyphae. *Conidiophores* micronematous, cylindrical, hyaline to pale brown, smooth-walled, sometimes reduced to conidiogenous cells. *Conidiogenous cells* holoblastic, monoblastic, integrated, terminal, determinate, hyaline to pale brown, smooth-walled. *Conidia* 38–52 × 19–33 μm (*x* = 47 × 26 μm, n = 50), solitary, acrogenous, cheiroid, smooth-walled, yellowish-brown to light brown, with a basal connecting cell, consisting of 5–7 (mostly 6) rows of cells, euseptate, unseparated, each row with 8–9 cells, individual rows discoid, secession schizolytic.

Culture characteristics: Conidia germinated on PDA within 24 h. Germ tubes produced from both ends. Colonies on PDA reaching 20–30 mm diam. after 2 weeks at 25 °C in natural light, circular, wrinkled, with dense white to pale yellow mycelia in the middle, sparser towards the edge; in reverse yellowish brown in the middle, pale yellow at the entire margin.

Material examined: China, Sichuan Province, Chengdu City, Dujiangyan City, Qingcheng Mountain scenic spot, 103°28′36′′ E, 30°55′9′′ N, on dead twigs of medicinal plant *Periploca forrestii*, 27 March 2021, H.Z. Du, S172 (HUEST 23.0213), living culture UESTCC 23.0213.

Notes: *Aquadictyospora lignicola*, the type species of *Aquadictyospora*, was introduced by Li et al. [5] from submerged decaying wood. In this study, an isolate was obtained from *Periploca forrestii*. Our collection (HUEST 23.0213) is similar to *A. lignicola* (MFLU 17-1422) in having yellowish-brown to light brown conidia, composed of 4–7 compactly arranged rows of cells, with a basal hyaline cell. Phylogenetic analysis showed that our strain clustered with the type strain of *A. lignicola* (MFLUCC 17-1318) with ML/BI 100%/1 bootstrap support (Figure 2). Thus, we identified our collection as *A. lignicola* and report it as a new host record.

**Figure 4:**
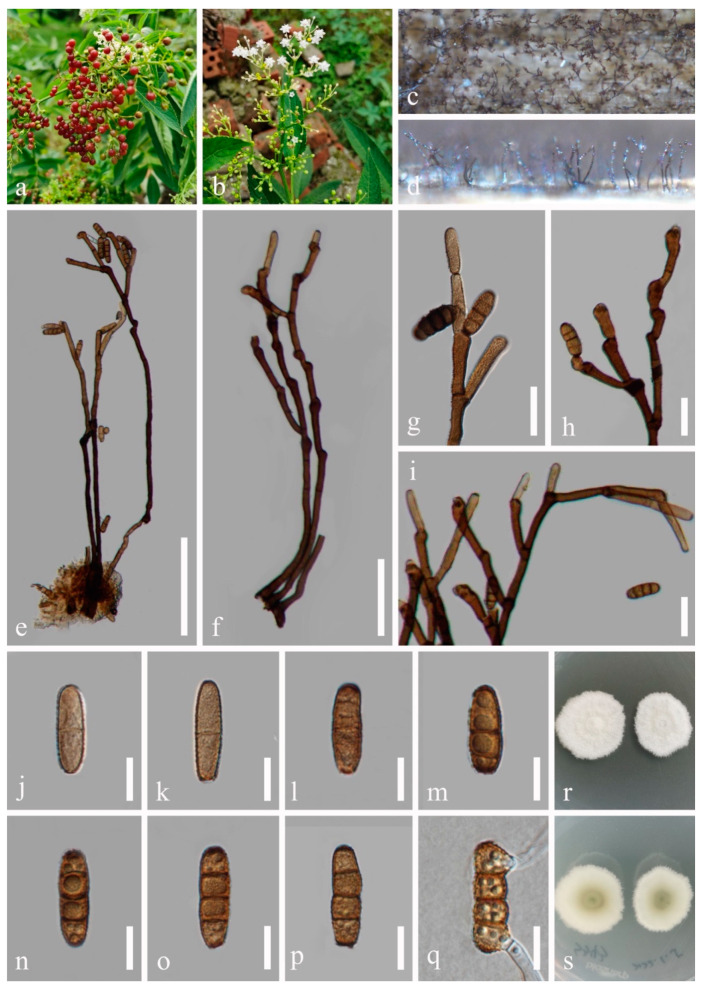
*Dendryphiella eucalyptorum* (HUEST 23.0214, new host record). (**a**,**b**) Host. (**c**,**d**) Colonies on a woody substrate. (**e**,**f**) Conidiophores. (**g**–**i**) Conidiogenous cells and conidia. (**j**–**p**) Conidia. (**q**) Germinated conidium. (**r**,**s**) Colonies on PDA, from above (**r**), from below (**s**). Scale bars: (**e**) = 100 μm, (**f**) = 50 μm, (**g**–**i**) = 20 μm, (**j**–**q**) = 10 μm.

***Dendryphiella eucalyptorum*** Crous & E. Rubio, Persoonia 32: 231 [24] (Figure 4).

Index Fungorum number: IF808918; Faces of Fungi number: FoF06712

*Saprobic* on dead twigs of *Sambucus javanica* Blume. Sexual morph: Not observed. Asexual morph: Hyphomycetous. *Colonies* on natural substrate superficial, effuse, dark brown. *Mycelium* mostly immersed, composed of smooth, septate, branched, brown hyphae. *Conidiophores* 185–300 μm long, 3–4 μm wide, macronematous, mononematous, brown, wider at the base, slightly paler at the apex, fasciculate, thick-walled, erect, straight or slightly flexuous, smooth or verruculose, septate, unbranched or sometimes branched, wider at the base. *Conidiogenous cells* 12–30 μm long, 5–7 μm wide (*x* = 20 × 6 μm, n = 30), polytretic, integrated, terminal and intercalary, later becoming subterminal, proliferating asymmetrically, clavate, brown, enlarged at the vertex. *Conidia* 12–27 × 5–7 μm (*x* = 21 × 6 μm, n = 50), subcylindrical, apex obtuse, base bluntly rounded, pale brown, aseptate or 1-septate when young, brown or dark brown, 3-septate when mature, constricted at septa, thick-walled, verruculose to verrucose.

Culture characteristics: Conidia germinated on PDA medium within 24 h. Germ tubes produced from both ends. Colonies on PDA reaching 10–15 mm diam. after two weeks at 25 °C in natural light, circular, cottony, with regular margins, white from above and white to pale grey from below.

Material examined: China, Sichuan Province, Chengdu City, High-tech West District, Yaobo Park, 103°56′21′′ E, 30°43′57′′ N, on dead twigs of medicinal plant *Sambucus javanica*, 11 August 2021, H.Z. Du, S343 (HUEST 23.0214), living culture UESTCC 23.0214; *ibid*., Leshan City, Muchuan County, Huangdan Town, 103°41′37′′ E, 29°12′58′ ′N, on dead leaves of medicinal plant *Ophiopogon japonicus* (L. f.) Ker-Gawl., 31 October 2021, H.Z. Du, S463 (HUEST 23.0215), living culture UESTCC 23.0215; *ibid*., Yaan City, Mingshan District, Wangu Town, 103°7′57′′ E, 30°10′45′′ N, on dead twigs of medicinal plant *Leonurus japonicus* Houttuyn, 29 October 2021, H.Z. Du, S459 (HUEST 23.0216), living culture UESTCC 23.0216.

Notes: *Dendryphiella eucalyptorum* (CBS 137987) was collected from *Eucalyptus globulus* in Spain [24]. We identified our collections as *D. eucalyptorum* based on morphology and phylogeny, and report them as new host records from medicinal plants (*Leonurus japonicus*, *Ophiopogon japonicus* and *Sambucus javanica*).

**Figure 5:**
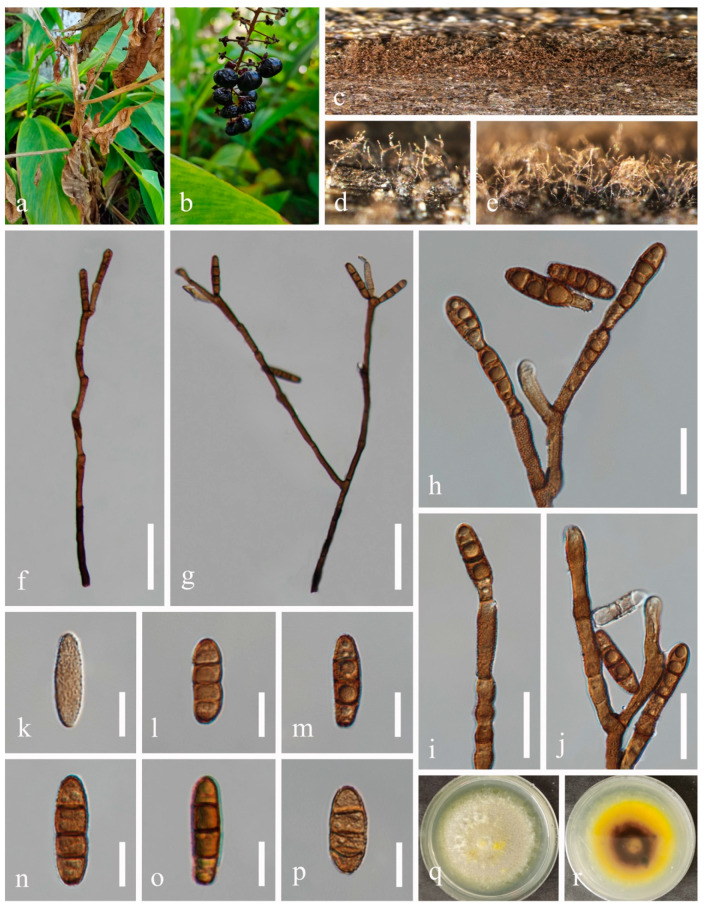
*Dendryphiella vinosa* (HUEST 23.0217, new host record). (**a**,**b**) Host. (**c**–**e**) Colonies on a woody substrate. (**f**,**g**) Conidiophores. (**h**–**j**) Conidiogenous cells and conidia. (**k**–**p**) Conidia. (**q**,**r**) Colony on PDA, from above (**q**), from below (**r**). Scale bars: (**f**,**g**) = 50 μm, (**h**–**j**) = 20 μm, (**k**–**p**) = 10 μm.

***Dendryphiella vinosa*** (Berk. & M.A. Curtis) Reisinger, Bull. trimest. Soc. mycol. Fr. 84(1): 27 [47] (Figure 5).

Index Fungorum number: IF329796; Faces of Fungi number: FoF08673

*Saprobic* on the twigs of *Phytolacca americana* L. Sexual morph: Not observed. Asexual morph: Hyphomycetous. *Colonies* on natural substrate superficial, effuse, brown to dark brown. *Conidiophores* 180–230 μm long, 4–7 μm wide, macronematous, mononematous, erect, straight or slightly flexuous, smooth or verruculose, septate, unbranched or sometimes branched, wider at the base. *Conidiogenous cells* 12–28 μm long, 5–7 μm wide (*x* = 18 × 6 μm, n = 30), polytretic, terminal, proliferating asymmetrically, brown, verrucose, enlarged at the vertex. *Conidia* 19–30 × 6–8 μm (*x* = 25 × 7 μm, n = 50), fusiform to ellipsoidal, pale brown to brown or dark brown, aseptate when young, 3-septate when mature, constricted at septa, thick-walled, smooth or occasionally verruculose.

Culture characteristics: Conidia germinated on PDA medium within 24 h. Germ tubes produced from both ends. Colonies on PDA reaching 40–50 mm diam. after two weeks at 25 °C in natural light. Mycelium superficial, with regular margins, white from above, and brown to yellowish in the middle zone from below, paler toward the margins.

Material examined: China, Guizhou Province, Guiyang City, Huaxi District, 106°39′59′′ E, 26°30′14′′ N, on dead twigs of medicinal plant *Phytolacca americana*, 24 January 2021, H.Z. Du, S61 (HUEST 23.0217), living culture UESTCC 23.0217.

Notes: *Dendryphiella vinosa* was initially isolated from unidentified rotten leaves by Reisinger [47]. It has also been reported on decomposing leaves in Japan [48] and *Dendrobium officinale* in China [49]. We identify our isolate as *D. vinosa* based on morphology and phylogenetic evidence. Therefore, we report *D. vinosa* from *Phytolacca americana* as a new host record in China.

**Figure 6:**
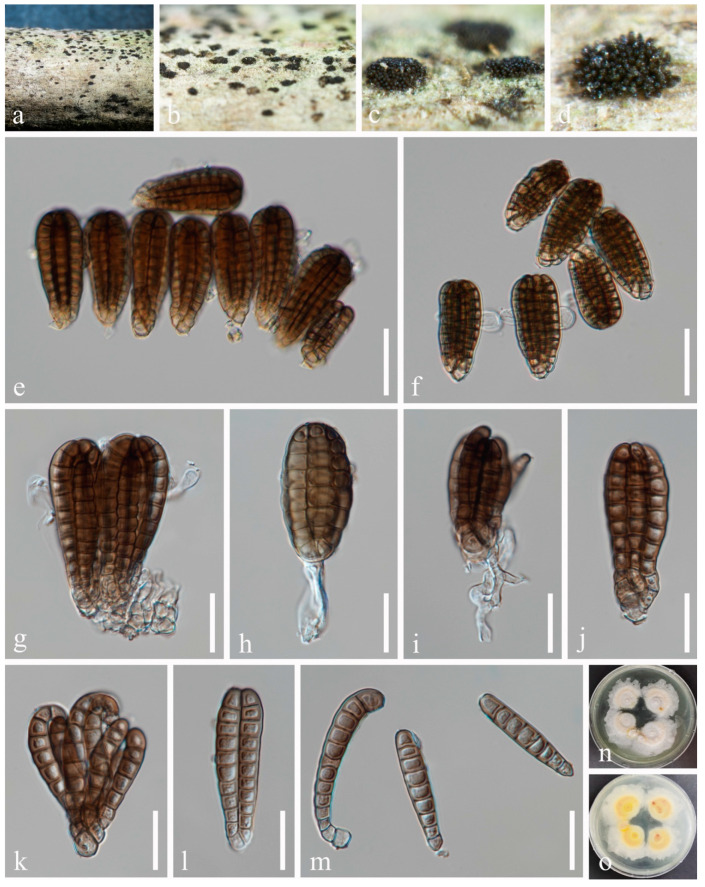
*Dictyocheirospora alangii* (HKAS 131314, holotype). (**a**–**d**) Colonies on a woody substrate. (**e**,**f**) Squash mount of a sporodochium. (**g**–**i**) Conidia with partial conidiophores. (**j**) Conidium. (**k**) Squashed conidium. (**l**,**m**) Arms of conidia. (**n**,**o**) Colonies on PDA, from above (**n**), from below (**o**). Scale bars: (**e**,**f**) = 30 μm, (**g**–**m**) = 20 μm.

***Dictyocheirospora alangii*** H.Z. Du, N. Wu & Jian K. Liu, sp. nov. (Figure 6).

Index Fungorum number: IF902300; Faces of Fungi number: FoF16017

Etymology: The epithet ‘*alangii*’ refers to the host genus *Alangium* from which the fungus was collected.

Holotype: HKAS 131314

*Saprobic* on dead twigs of *Alangium chinense* (Lour.) Harms. Sexual morph: Not observed. Asexual morph: Hyphomycetous. *Colonies* on natural substrate were punctiform, solitary, sporodochial, scattered, dark brown to black. *Mycelium* immersed, composed of pale brown, smooth, septate, branched hyphae. *Conidiophores* micronematous, short, branched, hyaline to pale brown. *Conidiogenous cells* 8–13 μm long, 3–6 μm wide (*x* = 10 × 5 μm, n = 20), holoblastic, cylindrical, integrated, terminal, pale brown, smooth, thin-walled. *Conidia* 44–60 × 17–27 μm (*x* = 49 × 20 μm, n = 50), solitary, cheiroid, ellipsoid to cylindrical, not complanate, pale brown to brown, consisting of 6–7 rows of cells, closely appressed, with rows cylindrical, palmately divergent, each row composed of 8–12 cells, euseptate, slightly constricted at the septa, guttulate, smooth, sometimes with 1–2 hyaline, globose to subglobose appendages which are 10–15 × 7–10 μm, and mostly attached at the central part of two outer arms.

Culture characteristics: Conidia germinated on PDA medium within 12 h. Germ tubes produced from both ends. Colonies on PDA reaching 40–50 mm diam. after one month at 25 °C in natural light. Mycelium superficial, with irregular margins, white from above, and yellowish in the middle zone from below, paler toward the margins.

Material examined: China, Guizhou Province, Guiyang City, Wudang District, Xiangzhigou scenic spot, near freshwater stream, 106°55′22′′ E, 26°46′26′′ N, on dead twigs of medicinal plant *Alangium chinense*, 24 February 2021, H.Z. Du, S130 (HKAS 131314, holotype); ex-type living culture CGMCC 3.25622 = UESTCC 23.0218.

Notes: The phylogenetic analysis showed that *Dictyocheirospora alangii* (CGMCC 3.25622) nested within *Dictyocheirospora*, close to *Di. multiappendiculata* (KUNCC 22-10734) and *Di. suae* (KUNCC 22-12424) with good bootstrap support (94% MLBS, 0.99 PP) (Figure 2). *Dictyocheirospora alangii*, *Di. multiappendiculata* and *Di. suae* share similar morphology, having cheiroid, ellipsoid to cylindrical, pale brown to brown conidia with hyaline, globose to subglobose appendages. However, *Di. alangii* differs from *Di. multiappendiculata* and *Di. suae* based on the number and location of appendages. *Dictyocheirospora multiappendiculata* has 1–6 subapical appendages, *Di. suae* has 1–3 subapical appendages, and sometimes two ellipsoid appendages are closer together on the same side of the conidia or both sides, whereas *Di. alangii* has only 1–2 appendages, one on each side of the conidia, and located in the central cells of the outer cell-row rather than near the apex. Therefore, *Di. alangii* is introduced as a new species based on morphology and phylogeny.

**Figure 7:**
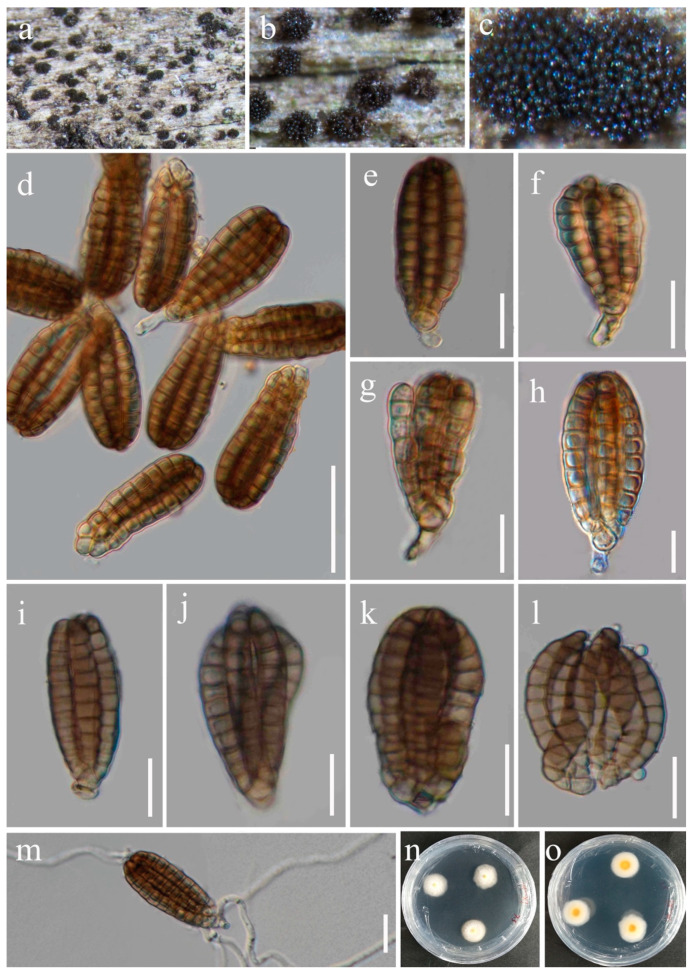
*Dictyocheirospora rotunda* (HUEST 23.0219, new host record). (**a**–**c**) Colonies on a woody substrate. (**d**) Squash mount of a sporodochium. (**e**–**h**) Conidia with partial conidiophores. (**i**–**l**) Conidia. (**m**) Germinated conidium. (**n**,**o**) Colonies on PDA, from above (**n**), from below (**o**). Scale bars: (**d**) = 50 μm, (**e**–**l**) = 30 μm, (**m**) = 20 μm.

***Dictyocheirospora rotunda*** M.J. D’souza, Bhat & K.D. Hyde, Fungal Diversity 80: 465 [2] (Figure 7).

Index Fungorum number: IF551581; Faces of Fungi number: FoF01262

*Saprobic* on dead twigs of *Euonymus japonicus* Thunb. Sexual morph: Not observed. Asexual morph: Hyphomycetous. *Colonies* on natural substrate scattered, scattered and dark brown. *Mycelium* composed of immersed or partly superficial, pale brown, smooth, thin-walled hyphae. *Conidiophores* micronematous, pale brown, smooth-walled. *Conidiogenous cells* holoblastic, integrated, terminal, cylindrical to subglobose, hyaline to pale brown, smooth, and thin-walled. *Conidia* 45–53 × 17–23 μm (*x* = 50 × 21 μm, n = 50), solitary, acrogenous, cheiroid, pale brown to brown, consisting of 5–7 rows of cells, rows digitate, cylindrical, inwardly curved at the tip, arising from a basal cell, without appendages, with each row composed of 10–12 cells, euseptate.

Culture characteristics: Conidia germinated on PDA medium within 24 h. Germ tubes produced from both ends. Colonies on PDA reaching 10–20 mm diam. after two weeks at 25 °C in natural light. Mycelium superficial, with regular margins, white from above, and brown to yellowish in the middle zone from below, paler toward the margins.

Material examined: China, Guizhou Province, Guiyang City, Huaxi District, 106°39′59′′ E, 26°30′14′′ N, on dead twigs of medicinal plant *Euonymus japonicus*, 24 January 2021, Y.R. Sun, CL30YR (HUEST 24.0200), living culture UESTCC 24.0183; *ibid*., Sichuan Province, Chengdu City, Dujiangyan City, Qingcheng Mountain scenic spot, 103°28′36′′ E, 30°55′9′′ N, on dead twigs of medicinal plant *Prinsepia utilis*, 27 March 2022, R.R. Liang, 123rui (HUEST 23.0219), living culture UESTCC 23.0219.

Notes: Boonmee et al. [2] introduced *Dictyocheirospora* with *D. rotunda* as the type species from decaying wood in Thailand. The new strains (UESTCC 23.0219 and UESTCC 24.0183) were collected from *Euonymus japonicus* and *Prinsepia utilis* in China. We identified our collections as *D. rotunda* (Figure 2) and report them as new host records from medicinal plants.

***Neoxylochrysis*** N. Wu & Jian K. Liu, gen. nov.

Index Fungorum number: IF902299; Faces of Fungi number: FoF16018

Etymology: “*Neoxylochrysis*” refers to the resemblance of its conidial morphology to the genus *Xylochrysis*.

*Saprobic* on *Typha latifolia*. Sexual morph: Not observed. Asexual morph: *Conidiomata* semi-immersed or immersed, visible as black shiny dots on the host, subcuticular in origin, then becoming erumpent, subglobose, brown to black, uniloculate solitary to scattered, glabrous, ostiolate. *Ostiole* single, centrally or laterally located. *Conidiomatal wall* comprising 4–5 layers, hyaline to dark brown, thick-walled cells of *textura angularis* in the upper part, becoming pale brown, thin-walled at the base. *Conidiophores* reduced to conidiogenous cells. *Conidiogenous cells* enteroblastic, phialidic, determinate, discrete, smooth-walled, aseptate, cylindrical to subcylindrical, or ampulliform, hyaline. *Conidia* hyaline, aseptate or 1-euseptate, without constrictions at the septum, oval or oblong to cylindrical, with rounded or obtuse ends, smooth, thin-walled, often with 2–4 small guttules in each cell, without sheath or appendages.

Type Species: *Neoxylochrysis typhicola* (Kamolhan, Banmai, Boonmee, E.B.G. Jones and K.D. Hyde) N. Wu and Jian K. Liu.

Life Mode and Known Distribution: *Neoxylochrysis* is reported as a saprobe on submerged stems of *Typha latifolia* in freshwater habitat. The genus is presently known from UK [50].

Notes: A new monospecific genus, *Neoxylochrysis*, is introduced herein to accommodate an asexual species in Dictyosporiaceae, typified by *N. typhicola*, based on distinct morphology and multi-locus phylogenetic analysis. *Neoxylochrysis typhicola* was previously described as *Pseudocoleophoma typhicola* by Hyde et al. [50] from submerged stems of *Typha latifolia* in freshwater environments from UK. *Neoxylochrysis* is phylogenetically related to *Pseudoconiothyrium* in the phylogenetic tree and formed an independent clade separated from *Pseudocoleophoma*. The asexual morph of *Neoxylochrysis* differs from the members of *Pseudocoleophoma* by having small, hyaline, oval-shaped, smooth, thin-walled, septate conidia with rounded or obtuse ends and guttules in each cell [50]. A comparison of the asexual morph of *Neoxylochrysis* with members of *Pseudocoleophoma* and intuitive photos of conidia are provided in Table 2. Since the sexual morph of *Neoxylochrysis* has not been discovered, it is impossible to compare the sexual morphologies of *Neoxylochrysis* with that of *Pseudocoleophoma*. Therefore, a new genus, *Neoxylochrysis*, is introduced in Dictyosporiaceae to accommodate a single coelomycetous species, *N. typhicola*.

**Figure 8:**
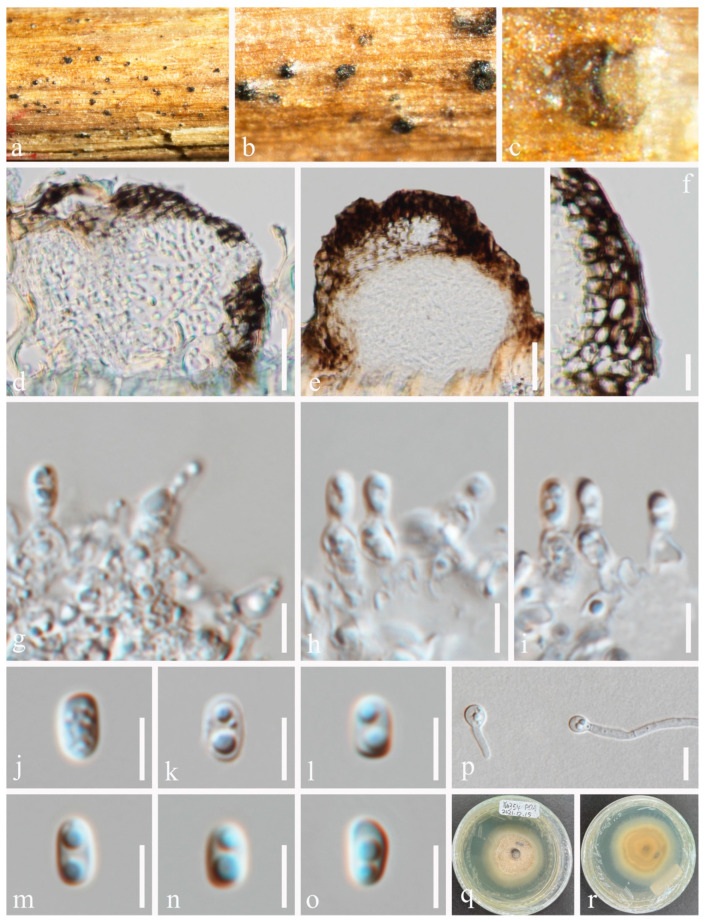
*Neoxylochrysis typhicola* (HKAS 131316). (**a**–**c**) Appearance of black conidiomata. (**d**,**e**) Vertical section of conidiomata. (**f**) Section of the peridium. (**g**–**i**) Conidiogenous cells and developing conidia. (**j**–**o**) Conidia. (**p**) Germinated conidium. (**q**,**r**) Colony on PDA, from above (**q**), from below (**r**). Scale bars: (**d**,**e**) = 20 μm, (**f**) = 10 μm, (**g**–**o**) = 5 μm, (**p**) = 10 μm.

***Neoxylochrysis typhicola*** (Kamolhan, Banmai, Boonmee, E.B.G. Jones and K.D. Hyde) N. Wu and Jian K. Liu, comb. nov. (Figure 8).

Index Fungorum number: IF902298; Faces of Fungi number: FoF16019

Basionym: *Pseudocoleophoma typhicola* Kamolhan, Banmai, Boonmee, E.B.G. Jones and K.D. Hyde, in Hyde et al., Fungal Diversity 80: 34 (2016) [50].

*Saprobic* on host plants, such as *Rhaphiolepis indica* and *Typha latifolia*. Sexual morph: Not observed. Asexual morph: *Conidiomata* 90–115 μm high × 130–150 μm diam. (*x* = 105 × 135 μm, n = 10), semi-immersed or immersed, visible as black shiny dots on the host, subcuticular in origin, then becoming erumpent, subglobose, brown to black, unilocular, glabrous, ostiolate. *Ostiole* 50–75 μm long, 70–100 μm wide, centrally or laterally located. *Conidiomatal wall* 20–25 μm wide (*x* = 23 μm, n = 20), comprising 4–5 layers, dark brown, thick-walled cells of *textura angularis*. *Conidiophores* reduced to conidiogenous cells. *Conidiogenous cells* 2.5–5.5 × 2–3 μm (*x* = 3.5 × 2.5 μm, n = 30), phialidic, smooth-walled, aseptate, hyaline. *Conidia* 7.5–11 × 2–3 μm (*x* = 10 × 2.5 μm, n = 30), hyaline, aseptate, oval, smooth, thin-walled, with 1(–2) guttules in each cell.

Culture characteristics: Conidia germinated on PDA within 12 h. Germ tubes produced from one end. Colonies on PDA reaching 30–35 mm diam. after two weeks at 25 °C in natural light. Mycelium superficial, grayish white from above, pale brown at the center, and yellowish white at the margin from below.

Material examined: UK, Hampshire, Swanick Lakes, on submerged stems of *Typha latifolia* in freshwater habitat, 28 August 2015, E.B.G. Jones, GJ190 (MFLU 16-0966, holotype; HKAS 94520 isotype), ex-type living culture, MFLUCC 16-0123, KUMCC 16-0007; China, Guizhou Province, Panzhou City, Wetland Park, 104°29′14′′ E, 25°39′33′′ N, on dead twigs of *Rhaphiolepis indica* in terrestrial habitat, 21 July 2022, Na Wu, YW354 (HKAS 131316), living culture, CGMCC 3.25688 = UESTCC 23.0221.

Notes: In our phylogeny (Figure 2), *Neoxylochrysis typhicola* (CGMCC 3.25688, MFLUCC 16-0123) clustered with *Pseudocoleophoma puerensis* (ZHKUCC 22-0204, ZHKUCC 22-0205) and *Pseudoconiothyrium broussonetiae* (CBS 145036), forming an independent clade in Dictyosporiaceae. This fungus was initially introduced by Hyde et al. [50] from submerged stems of *Typha latifolia* in freshwater, based on morphology and ITS and LSU sequence data. *Neoxylochrysi typhicola* can be distinguished from the above closely related species based on ITS and LSU base pair differences from *P. puerensis* by 76/557 bp (13.6%) in ITS and 17/841 bp (2.0%) in LSU, and *P. broussonetiae* by 55/557 bp (9.9%) in ITS, 15/841 bp (1.8%) in LSU. *Neoxylochrysi* is similar to *Pseudoconiothyrium*, *Pseudocyclothyriella* and *Xylochrysis* in their asexual morphs. However, *Xylochrysis* belongs to Woswasiaceae, whereas *Neoxylochrysi* belongs to Dictyosporiaceae. The genus *Neoxylochrysi* differs from *Pseudoconiothyrium* and *Pseudocyclothyriella* in having conidia that are hyaline, oval or oblong to cylindrical, rounded or obtuse ends, smooth, thin-walled, often with 2–4 small guttules in each cell, aseptate or 1-euseptate, not constricted at the septum, and without a sheath or appendages. The asexual morph of *P. puerensis* is undetermined and thus, the asexual morphologies of *P. puerensis* and *N. typhicola* could not be compared.

**Figure 9:**
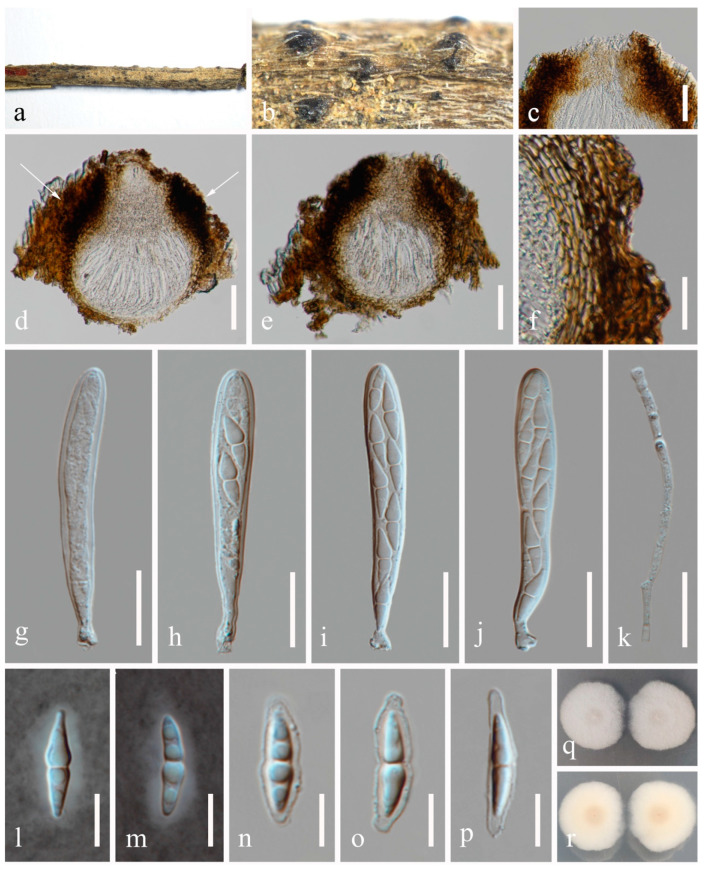
Sexual morph of *Pseudocoleophoma rosae* (HKAS 131315, holotype). (**a**,**b**) Appearance of thallus and ascomata on the host surface. (**c**) Vertical section through an ostiole. (**d**,**e**) Vertical section through ascomata. (**f**) Structure of peridium. (**g**–**j**) Asci. (**k**) Pseudoparaphyses. (**l**–**p**) Ascospores, (**l**,**m**) in Indian ink showing the sheath. (**q**,**r**) Colonies on PDA, from above (**q**), from below (**r**). Scale bars: (**c**,**f**–**k**) = 20 μm, (**d**,**e**) = 50 μm, (**l**–**p**) = 10 μm.

**Figure 10:**
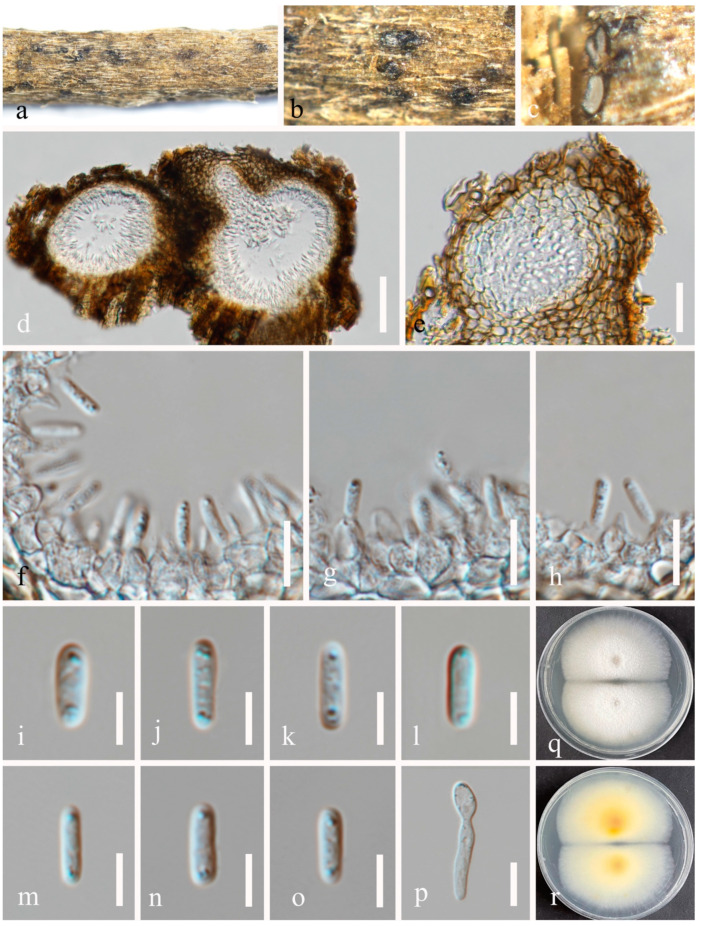
Asexual morph of *Pseudocoleophoma rosae* (HUEST 24.0201). (**a**–**c**) Conidiomata on the host substrate. (**d**) Vertical section of conidiomata. (**e**) Structure of the peridium. (**f**–**h**) Conidiogenous cells and developing conidia. (**i**–**o**) Conidia. (**p**) Germinated conidium. (**q**,**r**) Colonies on PDA, from above (**q**), from below (**r**). Scale bars: (**d**) = 50 μm, (**e**) = 20 μm, (**f**–**h**) = 10 μm, (**i**–**o**) = 5 μm, (**p**) = 10 μm.

***Pseudocoleophoma rosae*** N. Wu & Jian K. Liu, sp. nov. (Figure 9 and Figure 10).

Index Fungorum number: IF902297; Faces of Fungi number: FoF16020

Etymology: Referring to the host genus *Rosa* from which the fungus was collected.

Holotype: HKAS 131315

*Saprobic* on dead twigs of *Rosa roxbunghii* Tratt. Sexual morph: *Ascomata* 170–210 μm high × 175–220 μm diam. (*x* = 196 × 188 μm, n = 20), semi-immersed or immersed in the substrate, dark brown, globose to subglobose, unilocular, glabrous, thick-walled, thickened at the apex, visible as black dots or papilla on the host, ostiolate. *Ostiole* neck central, 59–71 μm long, 49–71 μm wide. *Peridium* up to 12–35 μm wide, composed of thick-walled, dark brown to pale brown or hyaline cells of *textura angularis*. *Hamathecium* up to 2–3 μm wide, hyaline, septate, branched. *Asci* 55–70 × 7–10 μm (*x* = 65 × 8 μm, n = 20), 8-spored, cylindrical to clavate, some slightly curved, with an ocular chamber, short-stalked with club-shape pedicel. *Ascospores* 12–20 × 3–6 μm (*x* = 16 × 4 μm, n = 50), hyaline, smooth-walled, fusiform with acute ends, 1-septate, slightly constricted at the septum, occasionally 2–4-guttulate when young, surrounded by a mucilaginous sheath; 1–2 μm wide at sides, 2–3 μm at each end. Asexual morph: Coelomycetous. *Conidiomata* 100–184 μm high × 98–215 μm diam. (*x* = 140 × 148 μm, n = 10), dark brown to black, visible as black dots covered by epidermal tissues, solitary to gregarious, globose to subglobose, pyriform or irregular in shape, unilocular, glabrous, ostiolate. *Ostiole* 30–40 μm long, 30–50 μm wide, cylindrical, centrally or laterally located. *Conidiomatal wall* 8–20 μm wide (*x* = 15 μm, n = 20), composed of thick-walled, dark brown to hyaline cells of *textura angularis*. *Conidiophores* reduced to conidiogenous cells. *Conidiogenous cells* 3–5 × 4–7 μm (*x* = 4 × 6 μm, n = 30), phialidic, aseptate, smooth-walled, hyaline. *Conidia* 6–9 × 2–4 μm (*x* = 8 × 3 μm, n = 50), hyaline, aseptate, oblong to cylindrical, smooth, thin-walled, guttules concentrated to ends.

Culture characteristics: Ascospores and conidia germinated on PDA within 12 h. Colonies on PDA reaching 30–40 mm diam. after two weeks at 25 °C in natural light. Mycelium superficial, with regular margins, slightly raised, fluffy, moderate aerial mycelium on the surface, underneath pale yellow.

Material examined: China, Sichuan Province, Leshan City, Huangdan Town, Muchuan County, 28°49′56′′ N, 103°40′40′′ E, on dead twigs of *Rosa roxbunghii*, 30 October 2021, Na Wu, H54 (HKAS 131315, holotype), ex-type living culture CGMCC 3.25623 = UESTCC 23.0220; *ibid*., H245 (HUEST 24.0201), living culture UESTCC 24.0184.

Notes: We isolated the asexual and sexual morphs of *Pseudocoleophoma rosae* from the same substrate, *Rosa roxbunghii*. The phylogeny indicates that these two isolates are identical (Figure 2). The multi-locus phylogenetic result (Figure 2) clearly showed that these two isolates are identical. And these two isolates constitute a distinct lineage but clustered close to *P. rhapidis* and *P. yunnanensis*. The sexual morph of *P. rosae* differs from *P. yunnanensis* by having smaller ascomata (170–210 × 175–220 μm vs. 160–280 × 200–280 μm), asci (55–70 × 7–10 μm vs. 65–90 × 8–11 μm) and ascospores (12–20 × 3–6 μm vs. 16–26 × 4–8 μm). Ascospores of *P. rosae* have guttules when young that disappear at maturity. The asexual morph of *P. rosae* differs from *P. rhapidis* by its smaller conidiomata (100–184 × 98–215 μm vs. 150–225 × 225–300 μm), narrower conidiogenous cells (3–5 × 4–7 μm vs. 7–10 × 13–17 μm), and smaller conidia (6–9 × 2–4 μm vs. 20–25 × 10–15 μm). In addition, the conidia of *P. rosae* have guttules concentrated at the ends, while *P. rhapidis* lacks guttules. Therefore, we introduce *P. rosae* as a new species based on the morphology and phylogeny.

## 4. Discussion

*Pseudocoleophoma* is a holomorphic genus distributed in Asia and Europe. Members of the genus have been reported as saprobes on various hosts and substrates in freshwater and terrestrial habitats, with no records as pathogens or endophytes [9,15,16,17,18,19,20,50]. Five *Pseudocoleophoma* species (*P. flavescens*, *P. paraphysoidea*, *P. rhapidis*, *P. rusci* and *P. zingiberacearum*) have been reported as asexual morphs, while four species (*P. guizhouensis*, *P. heteropanacicola*, *P. puerensis* and *P. yunnanensis*) are described based solely on their sexual morphs. Only *P. bauhiniae*, *P. calamagrostidis*, *P. polygonicola* and *P. rosae* are holomorphic species in *Pseudocoleophoma*. Morphologically, *P. calamagrostidis* and *P. polygonicola* have longer and narrower conidia than those of *P. typhicola*, which are wider and more rounded (Table 2) [9]. The location, size and number of the guttules also differ. In addition, the conidia of *P. calamagrostidis* and *P. polygonicola* have two small guttules concentrated at the ends of each cell, whereas *P. typhicola* has 2–4 large guttules filling the interior of the conidia. Also, the conidia of *P. typhicola* are septate or aseptate, whereas those of *P. calamagrostidis* and *P. polygonicola* remain aseptate throughout their entire growth cycle [9,50]. These characteristics have been confirmed in species, viz., *P. bauhiniae*, *P. rusci* and *P. zingiberacearum*, and their conidia are not similar to *P. typhicola*, but their morphology is similar to the type species, *P. calamagrostidis* [15,18,19].

When comparing the morphology of asexual species in *Pseudocoleophoma*, we not only found that the characteristics of *Neoxylochrysis typhicola* are different from other asexual species of *Pseudocoleophoma*, but also found that the characteristics of *P. flavescens* and *P. rhapidis* are different from *P. bauhiniae*, *P. calamagrostidis*, *P. polygonicola*, *P. rusci* and *P. zingiberacearum* [9,15,17,18,19,20,50]. Detailed characteristics and conidial sizes are shown in Table 2. We speculate that these differences are due to host and geographical factors.

*Pseudocyclothyriella clematidis* was initially introduced into *Pseudocoleophoma* by Phukhamsakda et al. [21] and placed between *P. calamagrostidis* (KT 3284) and *Neoxylochrysis typhicola* based on phylogenetic analysis. Jiang et al. [13] transferred *P. clematidis* to *Pseudocyclothyriella* based on morphology and multi-locus phylogenetic analysis. We observed that *N. typhicola* (MFLUCC 16-0123) and *Pseudoconiothyrium broussonetiae* (CBS 145036) also clustered in phylogenetic trees presented in two previous publications [6,27], separated from *Pseudocoleophoma*. As there were no fresh specimens, previous studies did not address the phylogenetic issues with *N. typhicola*. The results mentioned above coincide with our research. Fortunately, we re-collected *N. typhicola* from *Rhaphiolepis indica* (Rosaceae) in a terrestrial habitat. In morphology, our sample fits with the description of the *N. typhicola* holotype provided by Hyde et al. [50]. And our collection is phylogenetically identical to *N. typhicola*. The addition of fresh samples also allowed us to analyze the genus *Pseudocoleophoma* again, which confirmed that *N. typhicola* and *Pse. broussonetiae* clustered and solved issues related to the phylogeny of *N. typhicola*.

*Pseudocoleophoma puerensis* was introduced by Lu et al. [16] from a decaying branch of *Coffea arabica* var. *catimor* in China. Phylogenetic analysis showed that two strains of *P. puerensis* formed a distinct clade basal to all *Pseudocoleophoma* members, and it clustered with *Neoxylochrysis typhicola* [16]. *Pseudocoleophoma puerensis* only has a sexual morph, and it differs from *P. bauhiniae*, *P. calamagrostidis*, *P. polygonicola* and *P. yunnanensis* (Table 3)*. Pseudocoleophoma puerensis* does not belong to *Pseudocoleophoma*, but as no fresh samples of this species were collected, we retained it within the genus *Pseudocoleophoma* until new specimens are found.

The Dictyosporiaceae species investigated in this study were collected from ten different medicinal plants in Guizhou and Sichuan Provinces, China. These regions are characterized by a sub-tropical climate with favorable temperature and humidity conditions that support the growth of medicinal plants [51,52]. Fungal interactions with medicinal plants, including those involving endophytes [53,54], pathogens [55,56], and saprobes [57,58], are known to influence plant health and development. All of the taxa identified in this study were saprophytic fungi, and there is no evidence to indicate they negatively affect their host plants. In addition, our study suggests that the abundance of medicinal plants not only provides favorable conditions for the discovery of more microfungi, but also contributes to the diversity of microfungi associated with medicinal plants, offering a valuable reference for future studies on how fungi affect the potential medicinal value of these plants.

## Figures and Tables

**Table 1:** GenBank accession numbers and details of isolates chosen for the phylogenetic studies. The newly generated sequences are indicated in bold and ex-type strains are indicated with ^T^ after the strain number. N/A denotes no sequence available.

Taxa	Strain/Specimen Number	GenBank Accession Number			
		ITS	LSU	SSU	*TEF1-α*
*Aquadictyospora clematidis*	MFLUCC 17-2080 ^T^	MT310592	MT214545	MT226664	MT394727
*Aquadictyospora lignicola*	MFLUCC 17-1318 ^T^	MF948621	MF948629	N/A	MF953164
***Aquadictyospora lignicola***	**UESTCC 23.0213**	**PP925610**	**PP925664**	**PP925653**	**PP926517**
*Aquaticheirospora lignicola*	HKUCC10304 ^T^	AY864770	AY736378	AY736377	N/A
*Cheirosporium triseriale*	HMAS 180703 ^T^	EU413953	EU413954	N/A	N/A
*Dendryphiella eucalyptorum*	CBS 137987 ^T^	KJ869139	KJ869196	N/A	N/A
***Dendryphiella eucalyptorum***	**UESTCC 23.0214**	**PP925612**	**PP925666**	**PP925655**	**PP926520**
***Dendryphiella eucalyptorum***	**UESTCC 23.0215**	**PP925613**	**PP925667**	**PP925656**	**PP926518**
***Dendryphiella eucalyptorum***	**UESTCC 23.0216**	**PP925614**	**PP925668**	**PP925657**	**N/A**
*Dendryphiella phitsanulokensis*	MFLUCC 17-2513 ^T^	MG754400	MG754401	MG754402	N/A
*Dendryphiella trisepta*	COAD 2388 ^T^	MK278898	MK277357	N/A	N/A
*Dendryphiella vinosa*	MFLU 20-0444	MT907477	MT907480	N/A	N/A
***Dendryphiella vinosa***	**UESTCC 23.0217**	**PP925611**	**PP925665**	**PP925654**	**PP926519**
*Dictyocheirospora bannica*	KH 332 ^T^	NR_154039	NG_059061	NG_064841	AB808489
*Dictyocheirospora cheirospora*	KUMCC 17-0035 ^T^	MF177035	MF177036	MF928073	N/A
*Dictyocheirospora gigantica*	BCC 11346	DQ018095	N/A	N/A	N/A
***Dictyocheirospora alangii***	**CGMCC 3.25622**	**PP925607**	**PP925662**	**PP925652**	**PP926516**
*Dictyocheirospora multiappendiculata*	KUNCC 22-10734 ^T^	OP526632	OP526642	OP526623	OP542234
*Dictyocheirospora rotunda*	MFLUCC 14-0293 ^T^	KU179099	KU179100	KU179101	N/A
*Dictyocheirospora rotunda*	MFLU 18-1041	MH381764	MH381773	MH381758	MH388818
***Dictyocheirospora rotunda***	**UESTCC 23.0219**	**PP925608**	**PP925663**	**N/A**	**N/A**
***Dictyocheirospora rotunda***	**UESTCC 24.0183**	**PP925609**	**PP925661**	**PP925651**	**PP926515**
*Dictyocheirospora suae*	KUNCC 22-12424 ^T^	OP526631	OP526641	OP526622	OP542233
*Dictyocheirospora subramanianii*	BCC 3503	DQ018094	N/A	N/A	N/A
*Dictyocheirospora xishuangbannaensis*	KUMCC 17-0181 ^T^	MH388342	MH376714	MH388310	MH388377
*Dictyosporium elegans*	NBRC 32502 ^T^	DQ018087	DQ018100	DQ018079	N/A
*Dictyosporium olivaceosporum*	KH 375 ^T^	LC014542	AB807514	AB797224	AB808490
*Digitodesmium bambusicola*	CBS 110279 ^T^	DQ018091	DQ018103	N/A	N/A
*Gregarithecium curvisporum*	KT 922 ^T^	AB809644	AB807547	AB797257	AB808523
*Immotthia bambusae*	KUN-HKAS 112012AI ^T^	MW489455	MW489450	MW489461	MW504646
*Jalapriya pulchra*	MFLUCC 17-1683	MF948628	MF948636	N/A	MF953171
*Jalapriya toruloides*	CBS 209.65	DQ018093	DQ018104	DQ018081	N/A
*Neodendryphiella mali*	FMR 16561 ^T^	LT906655	LT906657	N/A	N/A
*Neodigitodesmium cheirosporum*	UESTCC 22.0020 ^T^	ON595714	ON595713	ON595712	ON595700
*Neoxylochrysis typhicola*	MFLUCC 16-0123 ^T^	KX576655	KX576656	N/A	N/A
***Neoxylochrysis typhicola***	**CGMCC 3.25688**	**PP925617**	**PP925671**	**PP925660**	**PP926523**
*Periconia igniaria*	CBS 845.96	LC014586	AB807567	AB797277	AB808543
*Periconia igniaria*	CBS 379.86	LC014585	AB807566	AB797276	AB808542
*Pseudocoleophoma bauhiniae*	MFLUCC 17-2586 ^T^	MK347736	MK347953	MK347844	MK360076
*Pseudocoleophoma bauhiniae*	MFLUCC 17-2280	MK347735	MK347952	MK347843	MK360075
*Pseudocoleophoma calamagrostidis*	KT 3284 ^T^	LC014592	LC014609	LC014604	LC014614
*Pseudocoleophoma flavescens*	CBS 178.93	N/A	GU238075	GU238216	N/A
*Pseudocoleophoma polygonicola*	KT 731 ^T^	AB809634	AB807546	AB797256	AB808522
*Pseudocoleophoma puerensis*	ZHKUCC 22-0204 ^T^	OP297799	OP297769	OP297783	OP321568
*Pseudocoleophoma puerensis*	ZHKUCC 22-0205	OP297800	OP297770	OP297784	OP321569
*Pseudocoleophoma rhapidis*	ZHKUCC 21-0124 ^T^	ON244664	ON244661	ON244667	ON243581
*Pseudocoleophoma rhapidis*	ZHKUCC 22-0004	ON244665	ON244662	ON244668	ON243582
***Pseudocoleophoma rosae***	**CGMCC 3.25623 ^T^**	**PP925615**	**PP925669**	**PP925658**	**PP926521**
***Pseudocoleophoma rosae***	**UESTCC 24.0184**	**PP925616**	**PP925670**	**PP925659**	**PP926522**
*Pseudocoleophoma rusci*	MFLUCC 16-1444 ^T^	MT185549	MT183514	MT214983	N/A
*Pseudocoleophoma yunnanensis*	ZHKUCC 22-0200 ^T^	OP297795	OP297765	OP297779	OP321564
*Pseudocoleophoma yunnanensis*	ZHKUCC 22-0201	OP297796	OP297766	OP297780	OP321565
*Pseudocoleophoma zingiberacearum*	NCYUCC 19-0052 ^T^	MN615939	MN616753	N/A	MN629281
*Pseudocoleophoma zingiberacearum*	NCYUCC 19-0053	MN615940	MN616754	N/A	MN629282
*Pseudocoleophoma zingiberacearum*	NCYUCC 19-0054	MN615941	MN616755	N/A	MN629283
*Pseudoconiothyrium broussonetiae*	CBS 145036 ^T^	MK442618	MK442554	N/A	N/A
*Pseudocyclothyriella clematidis*	MFLUCC 17-2177A ^T^	MT310595	MT214548	MT226667	MT394730
*Pseudocyclothyriella clematidis*	MFLU 16-0280	MT310596	MT214549	N/A	N/A
*Pseudodictyosporium elegans*	CBS 688.93 ^T^	DQ018099	DQ018106	DQ018084	N/A
*Pseudodictyosporium thailandica*	MFLUCC 16-0029 ^T^	KX259520	KX259522	KX259524	KX259526
*Sajamaea mycophila*	APA-2999 ^T^	MK795715	MK795718	N/A	N/A
*Verrucoccum spribillei*	SPO2343 ^T^	MT918780	MT918765	MT918773	N/A
*Vikalpa australiense*	HKUCC 8797	DQ018092	N/A	N/A	N/A

**Table 2:** Comparison of asexual morphs in *Pseudocoleophoma*.

Species	Morphology of Conidia	Conidiomata	Conidiogenous Cells	Conidia	References
*P. bauhiniae*	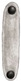	130–150 × 90–115 μm, immersed to superficial, subglobose, dark brown, multiloculate	2.5–5.5 × 2–3 μm, phialidic, hyaline, aseptate, doliiform to lageniform	7.5–11 × 2–3 μm, hyaline, oblong to ellipsoidal, aseptate, with guttules	[15]
*P. calamagrostidis*	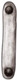	(150–)250–500 × 220–300 μm, immersed to erumpent, depressed globose, glabrous	5–9 × 2–4 μm, hyaline, doliiform to subglobose	6–10 × 2–2.5 μm, hyaline, cylindrical, aseptate, with guttules at each end	[9]
*P. flavescens*	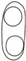	20–140 μm diam., globose, glabrous or covered by hyphae, solitary or confluent	4–6 × 3–6 μm, globose to doliiform	4–7 × 2–3.5 μm, hyaline, ellipsoidal, aseptate, with 2 very large polar guttules	[17,19]
*P. polygonicola*	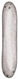	170–250 μm diam., superficial, ampulliform, glabrous, uniloculate	7–17 × 3.5–5 μm, hyaline, aseptate, doliiform to lageniform	(9–)11.5–18(–21.5) × 3–4.5 μm, hyaline, cylindrical, aseptate	[9]
*P. rhapidis*	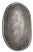	150–225 × 225–300 μm, immersed, subglobose, black, uniloculate	7–10 × 13–17 μm, phialidic, hyaline, doliiform	20–25 × 10–15 μm, hyaline, oblong to obovoid, aseptate	[20]
*P. rosae*	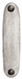	100–184 × 98–215 μm, semi-immersed, solitary to gregarious, globose to subglobose, pyriform or irregular, unilocular	3–5 × 4–7 μm, phialidic, aseptate, smooth-walled, hyaline	6–9 × 2–4 μm, hyaline, aseptate, oblong to cylindrical, thin-walled, guttules concentrated at the ends	This study
*P. rusci*	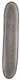	130–200 × 250–330 μm, deeply immersed, globose, subglobose or ovoid, brown, glabrous, unilocular	4–9 × 3–7 μm, phialidic, enteroblastic, hyaline, doliiform, ampulliform to subcylindrical	8–14 × 3–6 μm, hyaline, cylindrical or fusiform, with a rounded apex and a slightly narrow truncate base	[19]
*P. zingiberacearum*	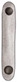	200–220 × 110–150 μm, immersed, multi-loculate, depressed globose, glabrous, non-ostiolate	1.5–2.5 × 1–1.5 μm, phialidic, doliiform to lageniform, hyaline, aseptate	12–14 × 2–3 μm, solitary, hyaline, aseptate, oblong to ellipsoidal, with rounded to obtuse ends, with guttules	[18]
*Neoxylochrysis typhicola* (*P*. *typhicola*)	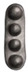	60–100 × 140–150 μm, semi-erumpent, uniloculate solitary to scattered, subglobose	2–5 × 2–5 μm, enteroblastic, smooth-walled, hyaline	9–11 × 2–3 μm, hyaline, oblong to cylindrical, with rounded or obtuse ends, septate, guttulate	[50], this study

**Table 3:** Comparison of sexual morphs in *Pseudocoleophoma*.

Species	Morphology of Ascospores	Ascomata	Asci	Ascospores	Reference
*P. bauhiniae*	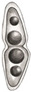	100–120 × 125–145 μm, immersed, subglobose to obpyriform, dark brown, coriaceous	65–80 × 5–8 μm, 8-spored, clavate to cylindric-clavate, short-pedicellate with an ocular chamber	17–20 × 3.5–4.5 μm, hyaline, cylindric-fusiform, 1–3-septate, 3–4-guttulate, without appendages and sheath	[15]
*P. calamagrostidis*	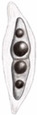	160–220 × 140–200 μm, immersed, globose to depressed globose	62.5–80 × 7.5–10 μm, 8-spored, cylindrical, rounded at the apex, short-stalked	(14.5–)16–19(–21) × 3–4.5 μm, hyaline, fusiform, 1-septate, 3–4-guttulate, with an entire sheath	[9]
*P. polygonicola*	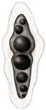	280–350 × 230–310 μm, immersed to erumpent	(67–)74–90(–100) × 9–12.5 μm, cylindrical to clavate	(17.5–)19–23(–25) × 4–6 μm, hyaline, fusiform, 1-septate, 4–6-guttulate, surrounded by a sheath	[9]
*P. puerensis*	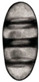	150–300 × 170–220 μm, immersed to erumpent, brown to black, globose or subglobose	50–70 × 7–11 μm, 8-spored, cylindrical, long-stalked with club-like pedicel	10–15 × 3.5–6 μm, hyaline to brown, narrowly ellipsoid or oblong, 1–3 thick and dark eusepta, normally 4-guttulate, without a sheath	[16]
*P. rosae*	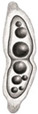	170–220 × 170–220 μm, semi-immersed or immersed, dark brown, globose to subglobose, unilocular, glabrous, thick-walled, thickened at the apex, visible as black dots or papilla on the host	70–88 × 8–12 μm, 8-spored, cylindrical to clavate, some slightly curved, with an ocular chamber, short-stalked with club-shaped pedicel	12–21 × 3–6 μm, hyaline, smooth-walled, fusiform with acute ends, 1-septate, slightly constricted at the septum, and occasionally 2–4-guttulate when young, surrounded by a mucilaginous sheath	This study
*P. yunnanensis*	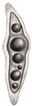	160–280 × 200–280 μm, semi-immersed to erumpent, dark brown to black, subglobose to obpyriform, solitary or scattered, coriaceous	65–90 × 8–11 μm, 8-spored, clavate to cylindrical, short-stalked with club-shaped pedicel	16–26 × 4–8 μm, hyaline, fusiform, 1-septate, 4-guttulate, with a distinct sheath	[16]

## Data Availability

All sequence data are available in NCBI GenBank with the accession numbers given in the manuscript.
